# Impact of traffic related air pollution indicators on non-cystic fibrosis bronchiectasis mortality: a cohort analysis

**DOI:** 10.1186/s12931-014-0108-z

**Published:** 2014-09-03

**Authors:** Pieter C Goeminne, Esmee Bijnens, Ben Nemery, Tim S Nawrot, Lieven J Dupont

**Affiliations:** Department of Respiratory Disease, University Hospital of Leuven, Herestraat 49, B-3000 Leuven, Belgium; Center for Environmental Sciences Hasselt University, Hasselt, Belgium; Department of Public Health and Primary Care, Laboratory of Lung toxicology, KU Leuven, Leuven, Belgium

**Keywords:** Non-cystic fibrosis bronchiectasis, Pollution, Traffic, Road, Mortality

## Abstract

**Background:**

Mortality in non-cystic fibrosis bronchiectasis (NCFB) is known to be influenced by a number of factors such as gender, age, smoking history and *Pseudomonas aeruginosa*, but the impact of traffic related air pollution indicators on NCFB mortality is unknown.

**Methods:**

We followed 183 patients aged 18 to 65 years with a HRCT proven diagnosis of NCFB and typical symptoms, who had visited the outpatient clinic at the University Hospital of Leuven, Belgium, between June 2006 and October 2012. We estimated hazard ratios (HR) for mortality in relation to proximity of the home to major roads and traffic load, adjusting for relevant covariables (age, gender, disease severity, chronic macrolide use, smoking history, socioeconomic status and *Pseudomonas aeruginosa* colonization status).

**Results:**

Fifteen out of the 183 included patients died during the observation period. Residential proximity to a major road was associated with the risk of dying with a HR 0.28 (CI 95% 0.10-0.77; p = 0.013) for a tenfold increase in distance to a major road. Mortality was also associated with distance-weighted traffic density within 100 meters (HR for each tenfold increase in traffic density 3.80; CI 95% 1.07-13.51; p = 0.04) and 200 meters from the patient’s home address (HR for each tenfold increase in traffic density 4.14; CI 95% 1.13-15.22; p = 0.032).

**Conclusion:**

Traffic-related air pollution appears to increase the risk of dying in patients with NCFB.

**Trial registration:**

The study was approved by the local ethical committee of the UZ Leuven, Belgium (ML-5028), registered at ClinicalTrial.gov (NCT01906047).

## Background

Both acute and chronic air pollution exposure have been shown to influence cystic fibrosis (CF) bronchiectatic disease. Ambient concentrations of ozone, PM_10_ and NO_2_ play a role in triggering an exacerbation in CF and annual average exposures to particulate air pollution was associated with an increased risk of pulmonary exacerbations and a decline in lung function [1,2]. Non-cystic fibrosis bronchiectatic (NCFB) disease is characterized by chronic bronchial inflammation caused by inappropriate clearance of various microorganisms and recurrent or chronic infections [3]. Mortality in NCFB is known to be influenced by a number of factors such as gender, age, smoking history and *Pseudomonas aeruginosa*, but data on the impact of air pollution on NCFB mortality are lacking [4,5]. More recently, De Soyza *and colleagues* formulated some research priorities in NCFB disease [6]. One of the three priorities is more research into epidemiology, to identify certain patient subgroups at risk for worse disease.

As the impact of air pollution in NCFB has not been studied before, our primary aim was to study the association between residential distance to a major road and death in a cohort of patients with NCFB. Secondary aims were to evaluate the distance-weighted traffic density at 100 and 200 meters from the patient’s residence.

## Methods

### Patient population

The study was based on the computerized notes of all patients between the age of 18 and 65 years who had a diagnosis of NCFB and had visited the outpatient clinic specifically dedicated to this condition at the University Hospital of Leuven, Belgium, between June 2006 and October 2012. Death was analyzed until end of November 2013. All patients had chronic productive cough and a chest HRCT scan confirming the diagnosis of NCFB [7]. All patients with a concomitant diagnosis of COPD were excluded from the analysis. HRCT scans were evaluated by two experienced radiologists. Images obtained using 1 mm collimation at full inspiration were reviewed and bronchiectasis was deemed to be present if one or more of the following criteria were fulfilled: a bronchoarterial ratio greater than 1, lack of tapering of the bronchi and visualization of bronchi within 1 cm of costal or paravertebral pleura or abutting the mediastinal pleura [7]. To further establish cause of the NCFB, each patient was evaluated with serum protein electrophoresis, immunoglobulins (IgA, IgE, IgM, IgG, IgG_1–3_), specific IgE and IgG against *Aspergillus fumigatus*, nasal NO measurement, sputum microbiology and an ororhinolaryngological evaluation. CF was excluded guided by clinical indication and using a sweat test, nasal potential difference, CF genotype screening or CF gene sequencing. Additional investigations were guided by clinical indication.

The following patient data were collected for analysis: home address at time of start of follow-up (which did not differ from home address at time of death), age, gender, smoking history, spirometric values at diagnosis (FEV_1_, FVC), type of bronchiectatic lesion (cylindrical, varicous or cystic), number of lobes affected, *Pseudomonas aeruginosa* infection status according to criteria by Lee *and colleagues* [8], chronic azithromycin use (a minimum dose of 250 mg three times a week during at least three consecutive months), socioeconomic status (SES: coded for each household and condensed into a scale with scores ranging from 1 (low) to 3 (high), taking into account occupation and the highest level of education reached by the households’ reference person or his/her partner [9-12]) and exacerbation frequency (recalled number of exacerbations in the year prior to inclusion; an exacerbation was defined as the need for antibiotic treatment due to respiratory symptoms). For all patients, death was assessed each six months in the patient file and if death occurred, the patient file was reassessed to identify cause of death. No patients were lost during follow-up.

### Distance-weighted traffic density

Previous research showed that airborne exhaust pollutants spread around a source in a Gaussian manner [13-15]. We observed a log linear association between survival and distance to major road. This means that the risk decreases more rapidly at shorter distances and flattens off at higher distances. In the construction of the distance-weighted traffic density analysis, we assumed the airborne spread around a source in a Gaussian manner and the impact of traffic was modeled as a function of the traffic intensity and the distance to the road according to the following equation:

$$ Y=\frac{1}{0.4*\sqrt{2\pi }} \exp \left[-\frac{1}{2}*\frac{{\left(D/150\right)}^2}{(0.4)^2}\right] $$

where D is the distance to the road and Y is the corresponding weight. The shape of the equation reflects the assumption that 96% of emissions disperse at 150 meters from the road, which is based on research that investigated the dispersion of vehicle exhaust pollutants [16-18]. We calculated distance-weighted traffic density in a buffer around each residential address by adding up the traffic intensity weighted by the distance. The resulting values were transformed logarithmically to normalize the distribution. Traffic intensity within a buffer was measured by the number of kilometers travelled by all vehicles during one day. The total length of the roads within each buffer was calculated using the Geographical Information System (GIS, ArcMap version 10.0). Information about the average number of vehicles on highways and other major roads was available from a network of measuring stations run by the Department of Mobility and Public Works of the Flemish Region. For minor local roads, no traffic counts were available. To include these traffic roads in our calculations, we made an estimation of the number of vehicles on the local roads. In order to estimate the number of vehicles on these non-major roads, we used information about their total length and the number of kilometers travelled on these roads in Flanders, which resulted in the average of 543 cars per day, a figure which was used in our calculations [19].

### Statistics

Cox proportional hazard regression analysis was used to estimate hazard ratios (HR) and the 95% confidence intervals (CI) for mortality in assocation to proximity of the home to major roads and traffic load, adjusting for relevant covariables (age, gender, smoking history, SES, disease severity, chronic azithromycin use and *Pseudomonas aeruginosa* colonization status). Analysis was performed using SAS 9.3. No a priori sample size calculations were done given the fixed study population.

### Ethical committee

The study was approved by the local ethical committee of the University Hospital Gasthuisberg, Leuven, Belgium (ML-5028) and the study was registered at ClinicalTrial.gov (NCT01906047).

## Results

### Patient characteristics

In total, we included 183 patients (55% female; 53 years, IQR 37–59), the majority of them having never smoked and having either an idiopathic (30%) or postinfectious (20%) cause of their bronchiectasis. There was a median follow-up time of 5.5 years (IQR 3.9-6.3). Over the study period, we registered 15 deaths. The majority of deaths had a direct respiratory cause (10 deaths; 67%; respiratory failure most often due to infection) or an admission due to respiratory problems causing further deterioration of the underlying disease status (one renal failure initially admitted for a severe respiratory infection/exacerbation; two non-respiratory end-stage tumors admitted due to severe pneumonia with further systemic complications and deterioration). Two patients had no clear respiratory cause for death or initial admission (one alcoholic patient died due to alcohol intoxication and cardiorespiratory arrest; one patient died of an unknown cause). Further patient characteristics are summarized in Table 1.

**Table 1:** **Patient characteristics**

**Number of patients**	183	
**Gender M/F**	45%/ 55%	
**Median age (years)**	53 [IQR 37–59]	
**FEV**_**1**_ **%**	73.2% ± 25.8	
**FVC%**	88.2% ± 25.3	
**Type of bronchiectasis**	Cylindrical 61.2% (n = 112)	
	Varicous 23.5% (n = 43)	
	Cystic 15.3% (n = 28)	
**n° of lobes affected**	One 20.8% (n = 38)	
	Two 26.2% (n = 48)	
	Three 29.5% (n = 54)	
	Four 15.8% (n = 29)	
	Five 7.7% (n = 14)	
**Exacerbations in previous year**	2 [IQR 1–3]	
**Socioeconomic status**	Low	51% (n = 93)
	Middle	46% (n = 84)
	High	3% (n = 6)
**Smoking history**	Never smokers	64% (n = 118)
	Passive smokers	2% (n = 3)
	Former smokers	19% (n = 35)
	Active smokers	15% (n = 27)
**Etiology**	Idiopathic	30% (n = 56)
	Postinfectious	20% (n = 37)
	PCD	16% (n = 29)
	Miscellaneous	13% (n = 23)
	Immunodeficiency	8% (n = 15)
	ABPA	7% (n = 12)
	Rheumatic	6% (n = 11)
***Pseudomonas aeruginosa*** **status**	Chronic colonization 12.5% (n = 23)	
**Deaths (events)**	15 events	
**Long-term antibiotic use**		
**- Azithromycin use**	56% (n = 103)	

Miscellaneous causes include sarcoidosis (5%), anatomic malformations (4%), inflammatory bowel disease (2%), aspiration (1%), vasculitis (1%). Deaths were due to respiratory failure (n = 10), kidney failure with a severe respiratory infection (n = 1), lymphoma with severe pneumonia (n = 1), pancreatic cancer with severe pneumonia (n = 1), alcohol intoxication with cardiorespiratory arrest (n = 1) and unknown (n = 1). ABPA = allergic bronchopulmonary aspergillosis; F = female; IQR = Interquartile range; M = male; PCD = primary ciliary dyskinesia; ± = standard deviation. Passive smokers were never smokers with high smoking exposure due to smoking peers or due to professional exposure. Exacerbations were the recalled number of antibiotic events in the year prior to inclusion.

### Pollution results

#### Unadjusted model

Unadjusted analysis showed that residential proximity to a major road was associated with the risk of dying, giving a HR of 0.47 (CI 95% 0.23-0.97; p = 0.042) for a tenfold increase in distance to a major road. In other words, living e.g. at 50 m from a major road was associated with a doubled risk (1 divided by 0.47) of dying compared to living 500 m away from a major road. Further analysis revealed a trend towards a significant association between the risk of dying and distance-weighted traffic density in a 100 meter buffer from patient’s home address (HR for each tenfold increase in traffic density 2.1; CI 95% 0.80-5.73; p = 0.13) with a similar trend for distance-weighted traffic density in a 200 meter buffer from the patient’s residence (HR for each tenfold increase in traffic density 2.3; CI 95% 0.82-6.27; p = 0.11).

#### Adjusted for age and gender

Adjusting for age and gender, residential proximity to a major road was associated with the risk of mortality, giving a HR 0.46 (CI 95% 0.22-0.96; p = 0.039) for a tenfold increase in distance to a major road. Analyzing the risk of dying and distance-weighted traffic density, a trend was seen for a 100 meter buffer from patient’s home address (HR for each tenfold increase in traffic density 2.1; CI 95% 0.77-5.68; p = 0.14) with a similar trend for distance-weighted traffic density in a 200 meter buffer from the patient’s residence (HR for each tenfold increase in traffic density 2.24; CI 95% 0.80-6.27; p = 0.12).

#### Adjusting for multiple factors

Adjusting for age, gender, chronic *Pseudomonas aeruginosa* status, smoking history, number of lobes affected, type of bronchiectatic lesion, FEV_1_, FVC, SES and macrolide therapy, residential proximity to a major road was associated with the risk of mortality, giving a HR of 0.28 (CI 95% 0.10-0.77; p = 0.013) for a tenfold increase in distance to a major road. In other words, living e.g. at 50 m from a major road was associated with an almost fourfold (1 divided by 0.28) higher risk of dying than living 500 m away from a major road. Using the same adjusting factors, a significant association between the risk of dying and the distance-weighted traffic density was found in a 100 meter (HR for each tenfold increase in traffic density 3.80; CI 95% 1.07-13.51; p = 0.04) and 200 meter buffer from patient’s home address (HR for each tenfold increase in traffic density 4.14; CI 95% 1.13-15.22; p = 0.032). Excluding the patient with the unknown cause of death and adjusting for previously mentioned covariates, results still showed a significant association between risk of dying and residential proximity to a major road (HR 0.26; CI 95% 0.09-0.74; p = 0.01). Similarly, no changes were perceived in the results for distance-weighted traffic density in a 100 meter (p = 0.036) and 200 meter (p = 0.035) buffer from the patient’s home address after exclusion of that patient.

## Discussion

Our analysis showed that residential proximity to a major road was linked to an increased risk of dying in patients with NCFB. Further analysis suggested that distance-weighted traffic density is an important factor in this risk.

We describe for the first time the impact of traffic related chronic air pollution exposure indicators in a NCFB population. With the exception of cystic fibrosis, there is a striking paucity of research into bronchiectasis [6]. For cystic fibrosis, chronic and acute air pollution exposure have been linked to exacerbations [1,2], but the impact on mortality has not been established yet. For other respiratory and cardiovascular diseases, research clearly indicates a pronounced negative effect on mortality. Air pollution triggers myocardial infarction [20], it is associated with cardiopulmonary mortality [21], and causes an increased risk of infant mortality [22]. In respiratory disease, it has been linked with lung cancer [21] and mortality or bronchiolitis obliterans syndrome after lung transplantation [23]. For COPD, an interquartile range elevation in black carbon concentrations is associated with a 7% increase in COPD mortality [24]. We now added NCFB to the increasing list of respiratory diseases where mortality is affected by traffic related air pollution.

To further strengthen our data, we also analyzed distance-weighted traffic density, and demonstrated a significant effect in a buffer of 100 and 200 meters from the patient’s residence. This is in line with previous literature showing that cardiopulmonary mortality is associated with living near a major road [25-27].

It is known that air pollution affects mortality in the general population. Carey *and colleagues* found that in their nationwide English cohort of 835,607 people, residential pollution concentration was associated with mortality with a HR of 1.02, 1.03 and 1.04 for PM_2.5_, NO_2_ and SO_2_ respectively. [28] Similar effect sizes of pollution in the general population have been seen by other groups [21,29], and are much lower than the effects we describe in our NCFB population. A possible reason might be the superimposed effect of air pollution on the vicious cycle of inflammation, infection and mucus plugging with proteolytic enzymatic actions. Whether air pollution also leads to a higher exacerbation rate, remains to be established.

These results again emphasize the ongoing need to sensitize policy makers in reducing traffic and air pollution [30]. Cesaroni *et al.* elegantly showed that a reduction in traffic in the city center of Rome not only effectively reduced air pollution, but also caused an average life gain of 3.4 days per person for the more than a quarter million residents living along the busy roads [31].

Our study has some limitations. The most important limitation is the low number of events. We aimed at investigating a hard endpoint in NCFB, i.e. death. However, NCFB is not a very lethal condition and our 15 deaths among 183 patients over the course of our study period are in line with the rates from literature. Loebinger *and colleagues* showed only 27 deaths among their 91 patients over a 13-year period (29.7%) [4]. This limited number of events forces us to be cautious when interpreting our results regarding the effect of pollution. We further tried to refine our exposure assessment by adding distance-weighted traffic density analysis. The association between mortality and traffic density within distances of either 100 or 200 meters from the patient’s home address, adds to the suggestion that there is a real effect of air pollution and traffic density on mortality.

On the other hand, a second limitation of our study might be overadjustment for covariates in our multivariate model. Correcting for many factors (gender, age, disease severity, SES, chronic macrolide use, chronic colonization by *Pseudomonas aeruginosa* and smoking history) when there are only few events might have led to overadjustment and therefore an incorrect estimation of the effect. On the other hand, the unadjusted model and the model where we only correct for age and gender resulted in similar significant effects. The largely similar results of both the adjusted and unadjusted models therefore suggest that this limitation probably had a limited effect on our overall outcome.

Thirdly, as one patient had an unknown cause of death, this death might be unrelated to air pollution. We preferred all-cause mortality as our primary outcome as this is a strong objective end-point which is not biased or influenced by confounders. When excluding this patient from the analysis, residential proximity to a major road was still associated with risk of dying as were the results for distance-weighted traffic density. A second patient had a cardiorespiratory arrest and alcohol intoxication. We included this patient as literature has shown that air pollution is known also to affect cardiovascular mortality [20].

In this analysis, we did not address seasonality and temporal changes. The traffic counts are based on annual average traffic counts on working days in 2010 and no information on seasonality is available. However, we believe that, as we studied a chronic outcome, even if a seasonal variation in traffic density would exist, the spatial variation is more important than potential temporal variation.

Concerns might be raised on changes in distribution of distance to a major road and traffic density. However, Pearson *et al.* previously showed that an increase in traffic over an eleven-year period still shows highly correlated densities [14].

Finally, for minor roads, the traffic intensity data was missing for some local roads and therefore these missing data were imputed with a default value of 543 vehicles per day. We believe that, as these roads were minor roads, measurement error with regard to defining busy and non-busy roads is likely small. This default setting is based on average counts on local roads and has been used in previous large studies on traffic related air pollution [32].

In conclusion, we found that living close to a major road is associated with a higher risk of dying in patients with NCFB. We interpret this as reflecting yet another adverse effect of air pollution.
